# Sensors for in situ monitoring of oral and dental health parameters in saliva

**DOI:** 10.1007/s00784-023-05206-9

**Published:** 2023-09-12

**Authors:** Julia Timpel, Stephanie Klinghammer, Leif Riemenschneider, Bergoi Ibarlucea, Gianaurelio Cuniberti, Christian Hannig, Torsten Sterzenbach

**Affiliations:** 1 Clinic of Operative and Pediatric Dentistry, Medical Faculty Carl Gustav Carus, Dresden University of Technology, Fetscherstraße 74, 01307 Dresden, Germany; 2 Else Kröner-Fresenius Center for Digital Health (EKFZ), Dresden University of Technology, 01309 Dresden, Germany; 3 Institute for Materials Science and Max Bergmann Center for Biomaterials, Dresden University of Technology, 01069 Dresden, Germany

**Keywords:** Sensors, Intraoral, Saliva, Real-time monitoring

## Abstract

**Objectives:**

The oral cavity is an easily accessible unique environment and open system which is influenced by the oral fluids, microbiota, and nutrition. Little is known about the kinetics and dynamics of metabolic processes at the intraoral surfaces. Real-time monitoring of salivary biomarkers, e.g., glucose, lactate, fluoride, calcium, phosphate, and pH with intraoral sensors is therefore of major interest. The aim of this review is to overview the existing literature for intraoral saliva sensors.

**Materials and methods:**

A comprehensive literature search was performed to review the most relevant studies on intraoral saliva sensor technology.

**Results:**

There is limited literature about the in situ saliva monitoring of salivary biomarkers. Bioadhesion and biofouling processes at the intraoral surfaces limit the performances of the sensors. Real-time, long-term, and continuous intraoral measurement of salivary metabolites remains challenging and needs further investigation as only few well-functioning sensors have been developed until today. Until now, there is no sensor that measures reliably beyond hours for any analyte other than glucose*.*

**Conclusions:**

Saliva’s complex and dynamic structure as well as bioadhesion are key challenges and should be addressed in the future developments. Consequently, more studies that focus particularly on biofouling processes and interferential effects of the salivary matrix components on sensor surfaces are required.

**Clinical relevance:**

By monitoring fluids in the oral cavity, as the entrance to the digestive system, extensive information can be obtained regarding the effects of foods and preventive agents on the oral microbiota and the tooth surfaces. This may lead to a better understanding of strategies to modulate oral and general health.

## Introduction

The oral cavity is a highly dynamic environment where all interactions are affected by the oral fluids, mainly saliva. Saliva is a complex body fluid that is easily accessible and, due to its properties, a reasonable non-invasive alternative to blood analysis [1]. Saliva is released by three salivary glands [2, 3] and multiple minor glands at the tongue and the palate [4]. Adults secrete around 600 mL of this fluid daily [5]. It permeates from the blood via transcellular or paracellular paths [1]. In addition, saliva is a detergent for teeth and the oral cavity [5] and has a pH of 6–7.5 [4, 6]. It mainly consists of water (99%) and contains multiple biomolecules, ions, and a unique microbiome with multiple microorganisms [5, 7–9]. The complex salivary proteome regulates the oral immune response as well as ions and signaling molecules [10]. Table 1 shows an overview of the salivary composition.

**Table 1:** Composition of saliva [5, 7–9, 28, 32, 131]

Components/metabolites	
Water	
Inorganic ions	K^+^, Na^+^, Ca^2+^, Mg^2+^, Cl^−^, F^−^, HCO_3_^−^, H_2_PO_4_^−^, HPO_4_^2−^ [9, 131]
Abundant functional proteins	> 2200 proteins (e.g., immunoglobulins, enzymes: amylase, lectin, glycoprotein, lysozyme, peroxidase, and lactoferrin) [9, 131, 132]
Lipids	Ammonia, fatty acids, triglycerides, neutral lipids, glycolipids, amino acids [9, 133, 134]
Hormones	Steroid hormones, cholesterol [9]
Mucins	MUC5B, MUC7, MUC19, MUC1, MUC4 [9, 135]
Glucose	[9]
Mucus	[131]
Urea	[9, 131]
Uric acid	[9, 131]
Microorganisms	> 700 [9]

In 2012, Wong published the Salivaomics Knowledge Base (SKB), where most information related to salivaomics (= microbiome, microRNA, transcriptome, metabolome, and proteome of saliva) were collected [11, 12].

Due to their excellent accessibility [5], salivary biomarkers are suitable for screening multiple unusual systemic conditions like diabetes [3, 13], gastro-esophageal disorders [14], ovulation of women [15], the uremic state in patients with chronic renal diseases [5, 16–18], virus infections [5], and different kinds of cancer [19]. More significant for dentistry, saliva can help assess oral cavity diseases like periodontal infections or is suitable for caries risk assessments [20]. Unlike blood parameters that are generally rather stable over shorter time periods, biomarkers in saliva often fluctuate within minutes and hours depending among others on consumption of food and drinks, oral care, or sleeping. Therefore, acquisition of single isolated samples often offers little information and might even be misleading. However, observing the kinetics of salivary parameters of interest can yield valuable data on clinical conditions and processes. For example, monitoring of metabolites (e.g., lactate) or temporary changes in pH after eating can mean important information about the risk of caries development. Therefore, monitoring of salivary parameters over longer time periods is generally superior to single sample acquisition. Hence, wearable intraoral sensors seem to be a promising tool to monitor such biomarkers.

The first attempts at intraoral sensor measurement were introduced in the 1950s when Thompson and Brudevold conducted time point measurements with micro-antimony electrodes safely placed in glass capillaries in probe tips for pH measurements [21]. One decade later, the monitoring of plaque pH with glass electrodes [22] and salivary fluoride with biochemical sensors [23] using partial dentures were successfully performed. Several sensors for measuring salivary parameters are established today, but no wearable sensor lasts on the market [24]. Recently, research groups faced the task of engineering wearable or continuous biosensor technology for saliva monitoring [25, 26], but different challenges hinder their success.

Due to its heterogeneity, saliva metabolite measurement is challenging as the presence of bacteria, epithelial cells, and leukocytes can affect proper monitoring [27]. Sampling standardization still seems to be an unsolved problem as the concentration of the ingredients depends on the flow rate, the gland, gender, time of the day, the stimulus, and several other factors [24, 27]. Thus, the variation in the sampling volumes and the influence of external factors like food and drinks is huge [28]. Furthermore, the wide range of saliva viscosity and, therefore, the dilution of compounds due to stimulation of salivary flow is a crucial problem when considering sensor development [24, 29]. Hence, it is essential for the design and placement of a wearable device that the fluid varies in consistency in different regions of the mouth [24]. Moreover, the influence of the temperature should be addressed when monitoring intraoral metabolites [30].

**Fig. 1:**
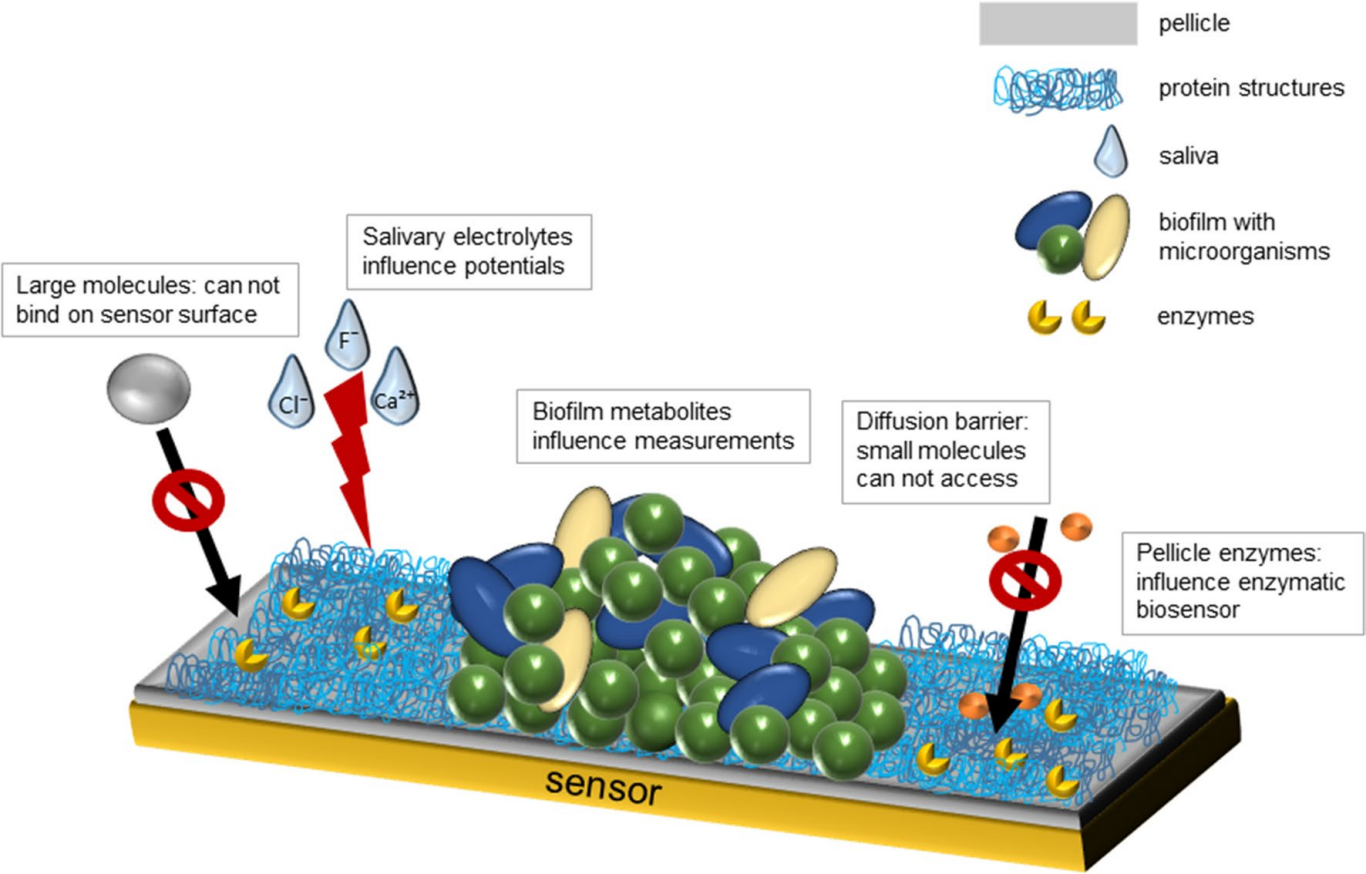
Simplified model of the bioadhesion and biofouling processes on sensor surfaces. The pellicle impairs larger molecules from binding directly on the sensor surfaces. It serves as a diffusion barrier which limits access of small molecules and ions on the sensor. Pellicle enzymes and metabolites from bacterial biofilms can influence measurements of the sensors, since they might interfere with underlying enzymatic reactions of biochemical sensors by either inhibiting or promoting these reactions. Salivary electrolytes might influence measurements in amperometric or potentiometric measurements due to their influence on conductivity. Biofouling and biodadhesion process can lead to worse performance of the intraoral sensor, which leads to a very low sensitivity of the sensor

The development of pellicle and biofilm within minutes on the surfaces of teeth, mucosal tissue, and sensors due to a high concentration of proteins like mucins and proteolytic enzymes in the saliva is a considerable challenge for intraoral sensor technology [1, 8, 28, 31, 32]. The pellicle is formed by selective adsorption of mainly salivary proteins, peptides, and other macromolecules from gingival crevicular fluid, bacteria, mucosa, and diet [33]. It serves as a lubricant and protects hard tissues against erosion. In addition, the pellicle contains many enzymes and biomolecules with antibacterial activity (e.g., lysozyme or amylase) [34]. Within a short amount of time, these surfaces then become colonized by microorganisms from the oral microflora, which can lead to the formation of biofilms [35]. Biofilm formation on surfaces in the oral cavity can cause various clinical conditions like caries [36], gingivitis [37], periodontitis [38], or periimplantitis [39]. The phenomenon of bioadhesion hampers conventional sensor surfaces for numerous ions and biomolecules to work correctly (Fig. 1). The pellicle impairs larger molecules from binding directly on the sensor surfaces. It serves as a diffusion barrier which limits access of small molecules and ions to the sensor [32, 40]. In addition, pellicle enzymes and metabolites from both bacterial biofilms and the host can affect measurements of the sensors, since they might interfere with underlying enzymatic reactions of biochemical sensors by either inhibiting or promoting these reactions [34]. Furthermore, especially ions, but also other substances, might influence measurements in amperometric or potentiometric measurements due to their impact on conductivity. The presence of ions might also influence the reference potential of electrodes. This biofouling process can lead to worse performance of the intraoral sensor, which, combined with low metabolite concentrations, leads to a very low sensitivity of the sensors [1, 28]. In some circumstances, pellicle formation on sensor surfaces might also be advantageous since it imitates the accessibility of metabolites to dental surfaces. A slightly acidic pH of the oral fluids does not mean necessarily an acidic pH directly at the tooth surface which would cause demineralization of enamel or dentine, respectively.

Another problem when measuring saliva in situ is the loss of data due to signal loss. For real-time monitoring of salivary parameters over several hours, the establishment of wireless communication and long-distance data transmission between the intraoral sensor and the readout device (e.g., smartphone) is still challenging [20]. Nowadays, the best and most energy-efficient strategies include the application of Bluetooth low energy (BLE) and near-field communication RFID (radio frequency identification) [41]. In addition, aspects like the potential toxicology of sensor materials, an impermeable encapsulation, and the development of wireless devices should also be kept in mind. Hence, transferring sensor technology to the oral environment is a significant challenge.

An optimal salivary sensor must fit appropriately to the anatomy of the mouth with minimal inconvenience to the wearer for long-term exposition [1, 42]. Furthermore, the device should be mechanically robust and securely fixed in the oral cavity [1, 20]. This anatomically correct incorporation of the sensors into the human mouth had considerable progress during the last decades. Therefore, sensor formats like flexible polymer foils on mouthguards [26, 43], pacifiers [42], dentures [23], vacuum-formed oral appliances [25, 30], or silk-based dental tattoos have been developed [44, 45].

Due to the many challenges of real-time measurements in saliva, knowledge about the dynamics and kinetics of salivary parameters is still scarce. Few research groups have faced the challenge of engineering wearable intraoral sensor devices. Therefore, this literature review focuses on those sensors measuring intraorally the most relevant salivary parameters for caries development and preventive dentistry.

## Methods

We performed a MEDLINE search using the following search terms: “saliva AND sensor”, “intraoral AND pH”, “intraoral AND glucose”, “intraoral AND lactate”, “intraoral AND calcium”, “intraoral AND phosphate”, “intraoral AND fluoride”. Moreover, we searched the reference lists of the included articles. For lactate, calcium, phosphate, and fluoride, we included studies describing sensor technology for salivary measurement of these molecules. Due to the extensive amount of studies referring to salivary glucose or pH sensors, we decided to specifically include articles for in situ monitoring of these parameters (see Table 2).

**Table 2:** List of different sensor technologies for the monitoring of the salivary parameters calcium, phosphate, fluoride, lactate, glucose, and pH

Sensor type	Publication
Target: Calcium	
* Continuous monitoring*	
Ion-selective electrodes	Nyein et al. [65]
Ion-selective electrodes	Lim et al. [63]
* PoC*	
Ion chromatography + piezoelectric sensors	Yu et al. [64]
Various lab equipment	Malik et al. [13]
Target: Phosphate	
* Real-time PoC*	
Enzymatic amperometric biosensor	Bai et al. [69]
* PoC*	
Enzymatic amperometric biosensor	Kwan et al. [75]
Potentiometric biosensor	Li et al. [74]
Electrochemical sensor	Chen et al. [76]
Fluorometric titration	Saikia and Iyer [48]
Colorimetric assays	Tobey and Anslyn [73]
Target: Fluoride	
* Real-time *in situ	
Potentiometric sensor	Graf and Mühlemann [23]
Potentiometric sensor	Clark and Dowdell [78]
* PoC*	
Colorimetric assay	Ghosh and Banerjee [60]
* Field water*	
Colorimetric sensor	Mukherjee et al. [79]
Target: Glucose	
* Real-time *in situ	
Electrochemical sensor	Arakawa et al. [25]
Electrochemical sensor	García-Carmona et al. [42]
* Real-time *in vitro	
Nanowire-based (FET) biosensors	Liu et al. [96]
* PoC*	
Electrochemical transistor	Ji et al. [93]
Colorimetric microfluidic paper-based mouthguard	de Castro et al. [92]
Electrochemical non-enzymatic sensors	Diouf et al. [94]
On-chip electrochemical sensors	Zhang et al. [91]
Target: Lactate	
* Real-time *in situ	
Wearable enzymatic amperometric sensor	Kim et al. [43]
* Real-time PoC*	
Wearable enzymatic amperometric sensor	Schabmueller et al. 103
Electrochemical transistors	Ji et al. [93]
* PoC*	
Cloth-based electrochemiluminescence	Yao et al. [102]
Electrochemiluminescence	Ballesta Claver et al. [109]
Cotton fabric-based electrochemical sensor	Malon et al. [2]
Target: pH	
* Real-time *in situ	
Glass electrode potentiometric sensor	Mühlemann and Graf [22]
Iridium oxide	Watanabe [115]
Antimony electrode	Ro et al. [117]
Wireless antimony electrode	Farella et al. [30]
In situ	
Hydrogel sensor	Tseng et al. [45]
* PoC*	
Optical sensor with PANI-gold hybrid nanostructures	Luo et al. [119]

## Technical background

The signal transduction mechanisms found in sensors for salivary analysis or for in situ monitoring in the oral cavity share the same ones typically found in wearable sensor technology (e.g., smart skins) [46]. Most of the published examples present optical, electrochemical, or electrical detection methods to identify and quantify biological or biochemical targets. The implementation of these methods can be achieved by employing low-cost materials and low power electronics, being appropriate for point-of-care (PoC) applications and personalized medicine.

**Fig. 2:**
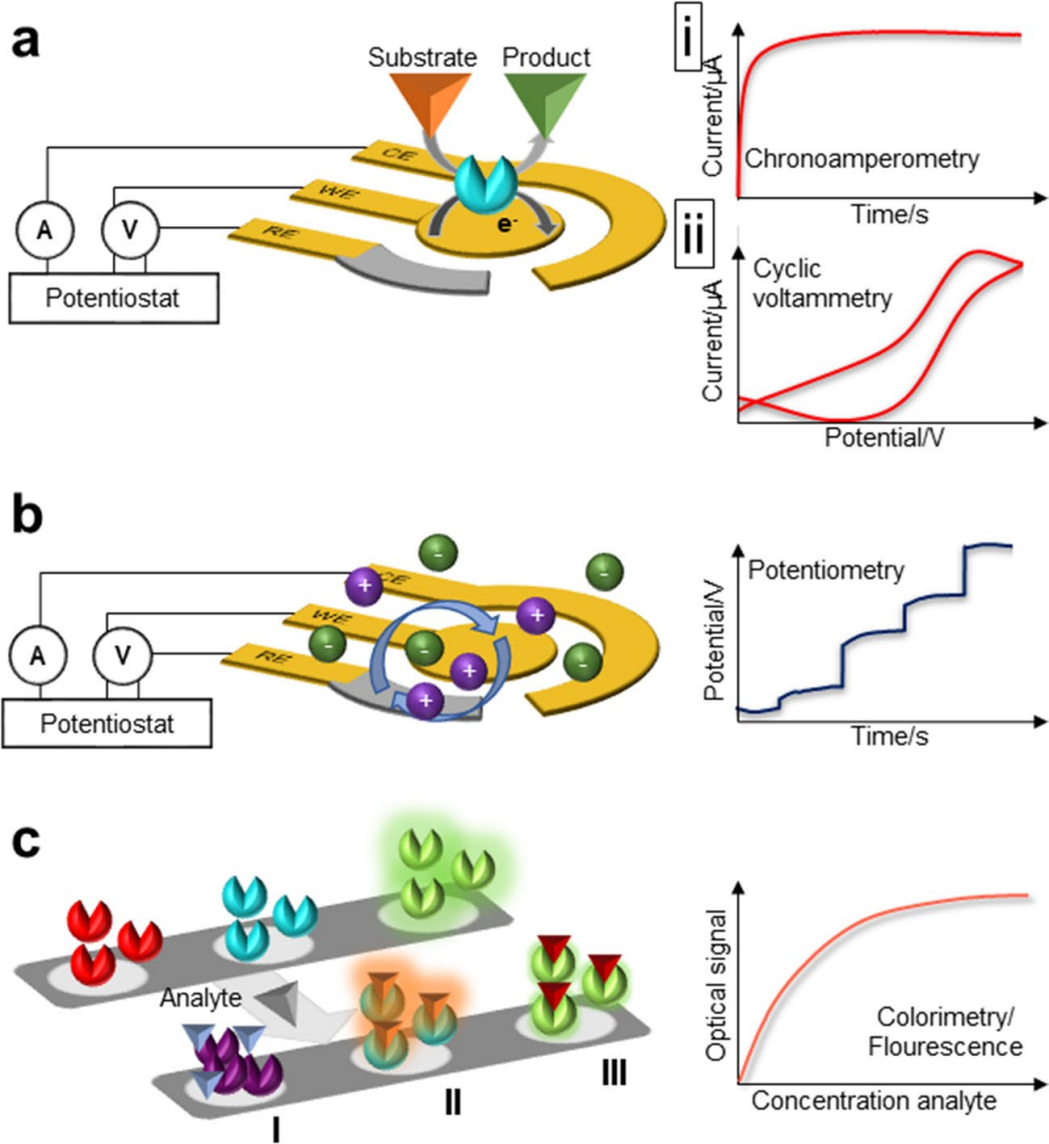
Schematic illustration of the most common biosensors suitable for detecting salivary analytes. **a** Optical biosensors monitor the reaction via optical signals such as absorbance, fluorescence, and luminescence. The sensors can operate either colorimetrically by inducing a measurable color change upon biorecognition with the analyte (I) or by fluorescence intensities, which are either enhanced (II) or quenched (III) after binding with the target analyte. **b** Amperometric biosensors monitor electrochemical reactions via measurable currents. Here, a current is produced when a potential is applied between two electrodes as a result of a redox reaction that can be followed resulting from the oxidation or reduction of the electroactive biological element involved. The rising current can be monitored as a function of time with an appropriate constant potential applied (chronoamperometry, panel i) or as a function of a varying potential that is swept between two values (cyclic voltammetry, panel ii). **c** Potentiometric biosensor monitors the reaction via measurable potentials. A charge potential at the working electrode is obtained and compared to the reference electrode when zero (or no significant current) is applied

Optical approaches such as fluorescence and colorimetry (Fig. 2a) provide relatively simple readout formats where even a naked eye readout is possible when quantification is not important. Fluorescence-based sensing is one of the most widely used bioanalytical techniques [47]. Fluorescence signal is based on the absorption of photons by a molecule at a given wavelength followed by emission at a longer wavelength. There are various mechanisms in which a sensor with such a transduction mechanism can work. For example, the fluorescence of a receptor present on a sensor surface can be quenched upon analyte binding, indicating the presence of the analyte in the sample [48]. An alternative mechanism would be adding labeled secondary receptors which will only attach to the sensor surface if the analyte is previously captured, providing the signal that evidences the analyte presence in the studied sample [49]. On the other hand, colorimetric sensors are used to detect analytes by simple color changes. For example, the presence of certain compounds can lead to the aggregation of nanoparticles, whose color change can be observed, e.g., silver nanoparticles reacting with volatile sulfur compounds in saliva [50]. When quantitative data needs to be obtained from optical sensors, the complexity of the overall architecture increases by the integration of light emitters and photodetectors or cameras and image analysis systems, making in situ measurements more difficult and limiting their use to in vitro detection [51].

In general, electrical or electrochemical transducers are capable of quantifying analytes in sensors with a simple format more suitable for the miniaturization, where multiple of them can be integrated in a single and small-footprinted chip for multiplexed analysis. Here, an electrical signal is altered depending on the concentration of the analyte in the biofluid. Different techniques can be found in this category as well. The dominant ones in the literature for in situ measurements are the amperometric and potentiometric sensors, due to their simplicity. Amperometric sensors (Fig. 2b-i) measure current changes at a constant potential, which can be originated for example from increased electron exchange due to the oxidation or reduction of an analyte [52]. In amperometric sensors, a set of three electrodes are only needed, namely the working, counter, and reference electrodes. The working electrode contains the necessary materials or receptor layers to make it specific to the target of interest, and the sensor measures the oxidation or reduction events taking place at its surface at a given potential with respect to the reference electrode. The counter electrode closes the circuit. With a slight increase in the required circuitry, a voltammetric sensor (Fig. 2b-ii) would scan the potential while measuring the current, showing signal peaks at the specific voltage levels where oxidation and reduction processes occur [53]. In certain cases, high-energy electron-transfer reactions can be produced at the electrode surface, generating light-emitting excited species, useful to develop electrochemiluminescence sensors [54]. However, their readout remains as a miniaturizing challenge for in situ sensing as in the case of the aforementioned optical sensors.

The same set of electrodes can also be operated as potentiometric transducers (Fig. 2c). In this case, the current flow between the working and counter electrodes is stopped as an open circuit (hence, open circuit potentiometry), and the potential variations (by comparing the working and the reference) due to the charging of the working electrode surface are considered (e.g., surface material protonation with sample acidification). The extremely low current levels in the system make it an advantageous nondestructive method [55]. By a further increase in the sensor complexity, field-effect transistors can be fabricated, which will also respond to the charging of its surface due to the presence of ionic species (protons, ions, biomolecules with a certain net charge, etc.). Here, two electrodes are connected with a semiconductor material, whose conductance can be controlled with the help of a gate electrode. The presence of ionic species nearby the semiconductor surface change the effective gate voltage, and in consequence, the conductivity will be affected [56]. Despite the higher complexity for sensor fabrication, ultrasensitivity is gained, with reported demonstrations down to the zeptomolar or attomolar levels [57, 58].

## Salivary ions in dentistry: calcium, phosphate, and fluoride

Enamel and dentin are composed of a significant degree of hydroxyapatite. The dissolution of hydroxyapatite due to nutrition and acidification leads to a local increase in salivary calcium and phosphate concentration [59]. This demineralization of enamel can subsequently cause caries or erosions on the tooth surfaces. Especially low molecular weight carbohydrates enhance caries progression, while frequent consumption of acidic drinks leads to dental erosions. Fluoride can partially counteract this demineralization of the enamel displacing the hydroxide ion in the enamel [60], leading to less calcium and phosphate increase [61]. Therefore, the concentrations of those three biomarkers are mandatory when measuring dental metabolites in the saliva. While the concentrations of calcium, phosphate, and fluoride in saliva are well-known for end-point measurements [6], few studies evaluated the kinetics of these parameters.

For the monitoring of calcium, phosphate, and fluoride ions, ion-selective electrodes are required as a subclass of electrochemical sensors [62]. All-solid-state ion-selective electrodes are one specific type of sensor classification that converts a specific ion’s activity to a measurable electrical signal [62]. This well-known type of wearable potentiometric sensor can be used to avoid a bulky detector system and hence is an optimal option for the intraoral measurement of ions [63]. Yet the technical implementation is still in progress, and until now, no device can detect relevant ions present in the oral fluids directly in the oral cavity [63, 64].

### Calcium

Calcium elevates in saliva not only due to dissolution from the enamel because of an acidic pH but also due to oral dehydration and increased protein concentration in diabetic patients [13]. Therefore, salivary calcium is an important parameter not just for oral but also for general health, which has to be in focus when thinking about real-time measurement of salivary parameters in a kinetic manner. The calcium concentration in resting saliva is 5.8 mg/100 mL, and in stimulated saliva, 6 mg/100 mL [6].

To our knowledge, there is no ion-selective wearable sensor available for measuring salivary calcium in situ in real time [65]. The reason is the tendency of calcium cations to create complexes to negatively charged sites of proteins or other metabolites [64]. Those interferences from other ions and ionic strength differences are difficult to analyze [64]. Furthermore, this reaction is pH-dependent after changing ionized calcium in the body fluids [66].

The most common approach for the continuous measurement of calcium ions in body fluids like sweat, tears, or urine is a wearable electrochemical sensor such as the one developed by Nyein et al. [65]. The application used an electrochemical-sensing platform to monitor calcium concentration by voltammetric measurements. The calcium-sensing electrode was fabricated from a thin organic membrane with electrically neutral carrier calcium ionophore II (ETH 129) and an ion–electron transducer (PEDOT: PSS). The monitoring was performed in situ and ex situ. The calcium sensitivity shows a near-Nernstian response with an average of 32.7 mV/decade [65]. Nyein and Lim used flexible ion-selective electrodes for potentiometric measurements of calcium. Monitoring those parameters can be carried out by maintaining the same electrode format but with a different surface modification incorporating specific ionophores [63, 65].

Other research groups performed salivary calcium measurements using ion chromatography, piezoelectric sensors, or customized ion-selective sensors as PoC devices [13, 64].

### Phosphate

There is limited literature about salivary phosphate (SP) biosensors, whereas many studies investigate sensors monitoring the phosphate concentration in water due to its enormous environmental implications [2, 67, 68]. Most of the approaches are not adaptable to intraoral applications due to the use of non-biocompatible materials like glutaraldehyde [67–70].

The high interest in SP for dental research arises from its essential role in enamel composition. SP can be used to predict the development of dental caries and the formation of dental calculus [71]. The concentration of phosphate in resting (16.8 mg/100 mL) and stimulated saliva (12 mg/100 mL) is higher than that of calcium with concentrations of 5.8 mg/100 mL in resting and 6 mg/100 mL in stimulated saliva, respectively [6]. Apart from the importance of SP for dental and oral considerations, the knowledge of the serum phosphate levels is essential for numerous health issues as the ovulation of women and the evolution during fertility treatment [15] or the uremic state in patients with chronic renal diseases [16–18]. As SP correlates with serum phosphate, monitoring the serum phosphate through SP levels is of interest [17, 18].

The standard methods for SP measurements are expensive, time-consuming, and onerous to the environment and health due to the usage of partially carcinogenic chemicals [72]. Several sensors were developed for SP assessment in recent years using different methods such as fluorometric titration [48] and colorimetric assays [73]; however, none of them showed reliable measurements with a broad sensor range and satisfying measuring time.

Li et al. monitored phosphate using a potentiometric detection method. They measured human saliva in aqueous solutions applying a sensor with ion exchanger-doped polymeric membrane electrodes as transducers. The implementation of metal complexes of 2,6-bis(bis(2-pyridylmethyl) aminomethyl)-4-methylphenol (H-BPMP) as receptors and catechols and their derivatives as promising potentiometric determinants resulted in good sensor selectivity and high sensitivity for phosphate ions in human saliva, urine, and mineral water [74].

In 2005, for the first time, Kwan et al. developed an amperometric biosensor for detecting phosphate in human saliva using pyruvate oxidase (PyOD) [75]. They immobilized PyOD on a screen-printed electrode by drop-casting a Nafion matrix (polymer) covered by a poly(carbamoyl) sulfonate hydrogel as a protective layer. Phosphate causes an enzymatic generation of hydrogen peroxide (H_2_O_2_) that can be monitored versus an Ag/AgCl electrode at + 420 mV. However, the saliva was collected from volunteers, and the measurement took place in vitro. Though, the sensor is recommended for oral/dental research due to its broad working range (7.5–625 µM), low detection limit (3.6 µM), and short response time that is required for one measurement (4 min). Furthermore, the measurement was efficient for 12 h, and 50 saliva samples showed a good correlation compared to commercial kits [75].

Recently, Bai et al. implemented a smartphone-based phosphate sensor that was capable of measuring artificial saliva in a reliable and reproducible way [69]. The research group used an inkjet-printed electrochemical enzyme-based biosensor system for the PoC detection of phosphate. Multi-walled carbon nanotubes were applied as an enzyme carrier to improve the enzyme loading. The cross-linking components were inkjet-printed layer-by-layer (BSA and glutaraldehyde) between printing layers on the screen-printed electrode. This combination improves reproducibility and allows customization. Reagents for the enzyme-catalyzed reaction were preloaded to achieve a portable system. The sensor showed a high selectivity and stability with a rapid response time (less than 10 s) and a wide linear range (160–2000 µM) for phosphate in artificial saliva using an amperometric measurement. When measuring artificial saliva, a recovery rate of 94% was achieved. Furthermore, a smartphone Android application for real-time data handling was developed [69]. The sensor showed good selectivity and stability due to glutaraldehyde as a cross-linker, which is not feasible for use in the oral cavity [69, 70].

Based on the hydration transition of magnesium hydrogen phosphate, Chen et al. developed an electrochemical sensor for SP monitoring. The sensor was prepared using a multi-walled carbon nanotube glassy carbon electrode modified with Nafion as a stabilizer. The surface was incubated in a magnesium phosphate solution and stabilized using Nafion. By applying differential pulse voltammetry vs. Ag/AgCl working electrode, a detection limit of 32 nM and a small working range (0.01 to 25 μM) were observed. Yet, the saliva samples were assessed in vitro and diluted 1500 times to decrease the phosphate concentrations into the linear range of calibration [76].

### Fluoride

Fluoride is the most important chemical element in preventive dentistry, leading to an incomparable decrease in caries development over the last decades [77]. However, the fluoride concentration in resting saliva is 0.028 mg/100 mL [6]. Indeed, the low concentration may cause problems for the development of sensors due to the necessity of a low detection limit.

Graf and Mühlemann performed the first in situ salivary fluoride monitoring. For this purpose, they used partial cobalt chrome dentures in which the potentiometric sensor was fixed. They successfully performed real-time measurements with limitations regarding the sensor size (power supply, transmitter) as it replaced some teeth in the denture. Furthermore, the risk of leakage of the internal fluoride-sensitive crystal (lanthanum fluoride) solution supposed a safety issue [23]. Clark and Dowdell tried a comparable approach facing similar problems regarding the exchange of the lanthanum fluoride solution [78].

In 2019, Ghosh and Banerjee developed a fluoride chemosensor for commercial use in regions that face naturally high fluoride concentrations in ground water. The research group developed the first fluoride sensor based on (E)-2-((2-(2,4-dinitrophenyl)hydrazono)methyl)-4-nitrophenol which was turned into a sensor test kit as a commercial product. They fabricated an electronic device that can turn chemical sensing signals into data output. For this, the patient had to spit in a vial, and the salivary fluoride caused a colorimetric change on the chemosensor, which was next synchronized by the electronic device into a readable output. Even though this sensor seems a big step toward salivary fluoride monitoring, it is not feasible for real-time monitoring as it is still a paper-strip based device for PoC diagnostics with a high affinity to high salivary fluoride concentrations [60].

Lately, Mukherjee et al. invented a smartphone-based fluoride-specific sensor for colorimetric detection of fluoride in water solutions at sub-ppm levels [79]. They synthesized core–shell nanoparticles with a chemoresponsive dye (xylenol orange) that was responsible for the rapid measurement. Unfortunately, the sensor is currently unsuitable for intraoral considerations due to the nanoparticles’ potential toxicity. Still, the study is noteworthy due to its “unprecedented” selectivity toward fluoride in the range of 0.1–5 ppm in field water samples. Furthermore, the research group conducted experiments on dental products like toothpaste and mouthwash. The sensor clearly showed a good performance regarding the detection limit, concentration, and reaction time compared to other reported sensors [79].

Even though the first salivary fluoride monitoring with biochemical sensors had already been performed successfully in the late 1960s [23], to our knowledge, there has been no sensor monitoring salivary fluoride in situ in real time until now. However, several noteworthy studies contributed to the development of sensors for fluoride monitoring in tap or field water [80–83]. Today, modified ion-selective electrodes with LaF_3_ crystals and FexOy nanoparticles seem to be the most common approach when considering miniaturizable intraoral application sensors [84]. LaF_3_ crystals are used for commercially available ionophore-based electrodes and are the state of the art when monitoring fluoride in watery solutions (e.g., DULCOTEST© by ProMinent®). Still, the in situ real-time measurement of salivary fluoride remains an open task.

## Salivary glucose measurement in situ

Glucose is one of the most commonly monitored biomolecules in body fluids. Therefore, multiple studies conducted in vitro or in situ experiments, mainly in blood, sweat, urine, and interstitial fluid but also in saliva [85–87]. As the salivary glucose (SG) concentration correlates to the blood glucose concentration, research on direct SG monitoring seems quite interesting for diabetes research [3]. For diabetic patients, using SG as a diagnostic tool is more than reasonable as an alternative and non-invasive screening method [28, 88]. Furthermore, SG plays an essential role in periodontal diseases where its elevation causes abnormal leukocyte chemotaxis, thickens the vascular basement membrane, and thereby reduces the anti-infective ability of periodontal tissues [3]. With high blood glucose levels, caries and periodontal disease risk increase and directly correlate with SG levels [89]. Furthermore, high SG levels increase the lactic acid concentration in plaque metabolism resulting in caries and periodontal infections [3]. Clark and Lyon fabricated the first glucose biosensor in the late 1960s [90]. Several research groups have focused on POC diagnostic devices for SG monitoring. Promising results using on-chip electrochemical sensors [91], microfluidic paper-based devices in mouthguards [92], electrochemical transistors [93], or electrochemical non-enzymatic sensors [94] can be found. The most common approach is SG sensing using enzymatic sensors with glucose oxidase (GOx) [13].

A mouthguard SG sensor for directly monitoring glucose concentration intraorally without pretreatment was demonstrated recently by Arakawa et al. [25]. The mouthguard consists of the sensing electrodes, the onboard electronics with Bluetooth low-energy (BLE) communication, and a transmitter. The sensor was integrated into the mouthguard, consisting of an anti-interference membrane (poly-dimethylsiloxane) and an enzyme membrane (MPC-co-EHMA) that facilitated immobilization for the GOx while offering suitable biocompatibility and mechanical strength. The GOx was attached on a poly(ethyleneterephthalate) glycol surface. A 200-nm-thick platinum working electrode and a 300-nm-thick Ag/AgCl reference electrode enabled amperometric measurements. The application was prepared with a layer of cellulose acetate film wrapped on the surface to eliminate the interference of ascorbic acid and uric acid in saliva. The sensor displayed a wide range for measuring SG concentration (20–200 μmol/L) and much more for glucose concentration in artificial saliva (1.75–10000 µmol/L). The device could directly transfer the data to a smartphone for output monitoring for about 20 min [25]. In 2016, the research group could set stable and long-term real-time monitoring (exceeding 5 h) with the developed telemetry system [95]. The approach could be a reasonable, high-efficient, non-invasive glucose detection method for diabetes in the future [3]. The device was further explored using a phantom jaw with a salivary flow system.

The first wearable sensor for real-time saliva sensing in newborns was developed by Garcia-Carmona et al., who used sensors in a pacifier form [42]. The sensing platform comprises wireless amperometric circuitry and a Bluetooth communication system for miniaturization and low-power operation. The sensor foil consisted of a flexible polyethylene-terephthalate (PET) sheet and an Ag/AgCl reference electrode. Furthermore, the electrochemical sensor was passivated to avoid material leakage into the newborn’s mouth. The group performed in situ measurements in real time with two individuals, comparing the SG before and after beverage intake for around 30 min. The sensor showed good stability and reproducibility and is a promising device for direct and real-time SG monitoring in newborns. In addition, the pacifier could significantly help to monitor SG in infants and neonates in neonatal intensive care units (NICUs). Currently, the device is limited by its operation time due to safety reasons and the short stability of the chitosan layer [42].

Another device for monitoring food consumption is a trilayer radio frequency tooth-mounted sensor consisting of a porous silk film and a PNIPAM hydrogel. Due to its flexible material, the sensor adheres to the tooth enamel and is an ideal temporary tattoo [44, 45]. The dielectric sensor of 2 × 2 mm in size with a thickness of 2.3 μm comprises biocompatible materials and can detect sugar, alcohol, pH, salinity, and temperature [45]. The sensor must be functionalized with analyte-sensitive layers: (a) a porous silk film and (b) a modified poly(N-isopropylacrylamide) hydrogel. The thin sensor has an active layer encapsulated between two split ring resonators and works without any battery. Additional wireless communication presents an excellent approach for wearable devices in intraoral monitoring. Hence, a self-powered solution is necessary to achieve the possibility of integrating the sensor into a dental tattoo [45]. In addition, those sensors can be easily multiplexed. The in situ use was demonstrated by adhering the sensor on a tooth following the consumption of tap water, apple juice, alcohol, mouthwash, and soup. In total, four tests were performed on patients. Data were recorded over a week by measuring deionized water, PBS, and glucose to examine the repeatability and stability of the sensors toward the various solutions.

Other groups investigated real-time glucose monitoring using nanoribbon- and nanowire-based field-effect transistor biosensors focused on artificial human saliva [96].

Although there are several approaches for monitoring glucose in sweat [97–101] or blood, research on in situ real-time SG analysis is still rare [3].

## Salivary lactate measurement

Lactate is a by-product of glucose metabolism whose elevation leads to a significant drop in pH and thus induces lactic acidosis [2]. Monitoring blood lactate is particularly important for critical care patients as acidosis can result in heart attacks [2, 102]. In addition, diabetic patients can benefit from lactate monitoring due to the close relationship between lactate and glucose metabolism [2]. Lactate diffuses passively from the blood to the salivary glands, where the concentration ranges from 0.1 to 2.5 mM [2, 103]. Salivary lactate (SL) correlates to blood lactate and is therefore an attractive alternative for non-invasive monitoring [28, 102]. However, lactate and other organic acids (e.g., acetate or pyruvate) are also produced within oral cariogenic biofilms and are a major cause for dental lesions and development of caries. This is mainly caused by glycolysis of glucose by *Streptococcus mutans*, *Lactobacillus*, or *Actinomyces* [104]. Moreover, lactate is an essential metabolite when monitoring physical stress and performance but also the intraoral initiation of digestion [28, 102, 105]. The lactate blood concentration arises during exercises as the musculature produces 40% of the circulating amount of lactate [106]. Hence, several sensors were reported to monitor lactate in the blood [107] or sweat [108], respectively.

Typically, lactate detection is conducted using electrochemical sensors [2, 26, 43, 103], colorimetric sensors [102], or electrochemiluminescence [102, 109]. In general, the mentioned methods show promising results but still suffer from high detection limits (> 0.1 mM), low sensitivity, large sampling volumes, complex preparation procedures, and high costs [102]. Most lactate sensors follow the same scheme as those for glucose, based on a specific enzyme (glucose or lactate oxidase) which oxidizes the analyte [2].

A wearable sensor concept for SL monitoring was invented by Schabmüller et al. [103]. The aim of the sensor was the intraoral real-time monitoring of SL during exercise and monitoring the results in a portable wireless manner. They created a miniaturized cavity-type sensor chip with a three-electrode configuration operating as a POC device. The sensor was equipped with a cavity and fine pores on its floor, in which lactate oxidase (LOx) was immobilized in a matrix of agarose gel and sealed by a self-adhering polyester foil. The lactate molecules diffuse through pores into this cavity, whereby the enzymatic reaction occurs following an amperometric readout [103]. The linear range of lactate measurement was 0.01–5 mM.

A wearable, non-invasive amperometric electrochemical sensor for the continuous monitoring of salivary metabolites was introduced by Kim et al. [43]. A three-electrode configuration sensor was screen-printed on a PET substrate and affixed to a mouthguard. Moreover, the sensor was based on a printable Prussian-blue (PB) transducer covered by a poly-orthophenylenediamine (PPD) that sealed the LOx reagent layer to avoid potential interferences. The sensor was recommended as a wearable sensor for continuous real-time lactate monitoring with a low detection potential (0.042 V versus Ag/AgCl) for the hydrogen peroxide formed. Lactate levels of 0.1–0.5 mM were detected with this device. The sensor was able to monitor salivary uric acid, too. A considerable benefit of the sensor is the permselective behavior of the PPD layer that minimizes the possibility of commonly present interferences with coexisting electroactive materials and proteins. Likewise, electropolymerized PPD is a good protection against biofouling in human saliva as it is commonly used to embed oxidases and to protect the biosensor surface. The sensor integrates the wireless data transmission capability to transmit data over long distances and continuously monitor SL for 2 h with a recurring measurement every 10 min [43]. Even though the sensor was fully wearable, the platform showed difficulties integrating the required electronics for continuous monitoring without connecting to an external analyzer. Therefore, the research group introduced a second version of the sensor to improve this deficiency [26]. However, the improved sensor was only functionalized for uric acid. The electronics were anatomically appropriate and miniaturized by featuring a potentiostat, a microcontroller, and a BLE transceiver. Accordingly, the device offered a sensitive, selective, stable, and rapid method to obtain dynamic data on salivary biomarkers in the oral cavity. Nonetheless, the invented mouthguard device was still bulky and not applicable for daily use, such as glucose monitoring in diabetes patients [28]. Moreover, no data were reported about the evaluation of the device under real-time in situ conditions.

Another sensitive electrochemical lactate sensor for PoC diagnostics was proposed by Ji et al. using chitosan for immobilizing the enzyme LOx and platinum nanoparticles to enhance the electrostatic forces. The sensor also worked for glucose monitoring using GOx. In addition, human saliva samples of healthy and diabetic patients were collected and successfully tested. Moreover, a prototype of a portable sensor device for real-time measurement was created, enabling a Bluetooth connection to a smartphone [93].

Other groups focused on the development of PoC devices using an electrochemiluminescence approach. For example, a SL sensor based on the enzymatic reaction of LOx and luminol was introduced by Ballesta Claver [109]. The disposable sensor showed a dynamic range of lactate detection from 0.1 to 0.5 mM. However, the saliva samples were diluted (1:4), filtered, and pretreated to oxidize ascorbic acid to reduce the effects of potential interferences from additional salivary components like uric acid. A cloth-based electrochemiluminescence SL biosensor with a smartphone-based readout was reported in 2017 [102]. The chemical reaction of luminol/H_2_O_2_ formed the base of a fast, accurate sensor and offered a low-cost option for in vitro salivary lactate PoC diagnostics.

A cotton fabric-based electrochemical SL sensor was introduced by Malon et al., who developed a device based on carbon electrodes functionalized with Prussian Blue and LOx [110]. The sensor offered a working range for SL of 0.1 up to 5 mM. The fabrication of the device was simple and inexpensive but had limitations due to the fluid flow in the cotton fabric platform, which occurred via capillary forces.

## Salivary pH measurement in situ

Considering the maintenance of a healthy intraoral status, evaluating the pH is of great importance as pH is a key factor for developing dental erosion and caries. The pH can decrease after intake of acidic foodstuff [111] or due to intrinsic acids related to gastro-esophageal disorders with regurgitation [14]. Typically, patients who have diabetes have a more acidic pH as a result of higher intraoral microbial activities that are in turn, caused by higher salivary glucose levels [13].

The pH of resting saliva ranges from 6 to 7.5 [6], depending on the time of the day and the time after food intake [30]. The critical pH for developing dental caries lies between 5.5 and 5.7 [111]. At lower pH values, the dissolution of enamel starts [111]. For erosion, the critical pH is 4.5 but without any plaque formation [30]. In addition, an acidic pH can point to a mineral deficiency or chronic generalized periodontitis, whereas an alkaline pH can signal plaque formation or generalized gingivitis [112]. The salivary pH can report a patient’s clinical status regarding diabetes, gastro-esophageal reflux, local inflammations, and infections [112].

In 1965, Mühlemann and Graf developed a method for in situ pH measurement of interdental plaque with a small glass electrode and radiotelemetry [22]. Likewise the fluoride sensor, the research group used partial dentures to replace extracted molars with a sensor. The potentiometric sensor used Ag/AgCl as a reference electrode and was fabricated by incorporating liquid junction-based electrodes. With the help of miniaturized transmitters and a power supply, the radio signal was received up to 2 feet. The device was the first approach using telemetry of plaque pH intraorally with a good accuracy of ± 0.1 of a pH unit. Mühlemann and Graf showed that the plaque pH on the sensor surface dropped significantly after glucose intake in a thick 5-day plaque. Even though the sensor monitored salivary pH continuously, there were several limitations. Glass electrodes have the disadvantage of being fragile and suffering from potential drifts [113]. In addition, replacing some molars was necessary, possible leakage of the internal solution could occur, and the need for a time-consuming calibration step contributed to the disadvantages of the sensor [22]. Furthermore, potentiometric sensors appeared to be temperature-dependent. Minamitani and co-workers faced the problem by adding a temperature sensor into a pH electrode and mounting this on a denture to keep the devices small [114].

Watanabe et al. developed an iridium oxide pH sensor with a telemetry device that evaluated pH values in a range of 2–12. The sensor was integrated into a full-mouth denture and had to be taken out during food intake. The system did not work appropriately for continuous high-resolution measurements as the range was limited to pH 5–9. Still, the device could monitor pH values over 7.5 h, with data acquisition every 4 s [115].

Ro et al. reported an antimony electrode for a continuous intraoral pH measurement up to 24 h. The wireless sensor was fully integrated into a splint buccally and could stay intraorally for 24 h without any leakage. However, the sensor itself limited the study as the principle of the electrode was to measure a voltage difference between a reference electrode and an antimony electrode. Besides, antimony is potentially toxic, depending on its oxidation [116]. Here, the electrode must be calibrated in buffer solution for 10 min before application to display the pH value. The detection range of pH was limited to 2–8.9 [117].

In 2016, a wireless intraoral model for the continuous measurement of pH and temperature in vitro and in vivo was presented [30]**.** A pH antimony electrode was developed for intraoral application and mounted into a splint that covered the whole dental arch and palate. Next, the sensor tip was placed palatally on the upper central incisors. Data were collected from eleven healthy participants for a time period of 24 h. The sensor showed good sensitivity compared to a standard glass electrode within pH ranges between 2.9 and 9.2. By placing the sensor intraorally, the maximum Bluetooth transmission distance to a smartphone was 1–2 m and accordingly yielded a data loss of 9–12.5%. The loss could be caused by an out-of-range location of the smartphone. The working group confirmed that there was no influence of the intraoral sensor position on the pH measurement. In addition, the experiments demonstrated that sleep influences the pH, which decreases during sleep, and the temperature, which enhances during sleep. The comparably big splint could inhibit the saliva flow rate [30].

PANI (polyaniline) membrane-based sensors are a well-established concept with the potential for wireless devices [118]. Luo et al. designed an optical sensor with PANI-gold hybrid nanostructures for monitoring pH in saliva with the potential for real-time monitoring [119]. The optical sensor surmounts electrochemical sensors for interferences that arise from ion electrolytes. The sensor showed a response time of 3.5 s, a linear pH response between 2 and 8, and stable measurements compared to a standard laboratory method [119].

An alternative approach for dental monitoring is the application of a dental tattoo based on a hydrogel sensor (see Glucose sensors) [45]. Here, the degree of the hydrogel’s swelling depends on the food beverage’s pH. A resonance frequency variation in the metal antenna inside the sensor is used to follow the swelling. Tseng et al. invented this sensor for food discrimination for people with diabetes or allergy sufferer.

In future context, using graphene-based flexible sensors for real-time pH sensing could be a worthwhile approach to improve pH monitoring. Graphene has excellent electrical, biochemical, and mechanical properties and is a promising candidate for dental sensors [120, 121].

## Discussion

Monitoring salivary metabolites and ions is a complex research field with unresolved challenges. Sensoring salivary dental parameters is of great importance for the prevention of oral diseases like caries or demineralization and remineralization on the tooth surfaces as well as monitoring of digestion processes in health an disease. Moreover, in situ saliva monitoring could help to get better knowledge of taste and taste perception in the oral cavity as well as the influence of different preventive dentistry approaches.

One of the main issues is the complexity of the oral fluids with its several components (see Table 1) and the formation of a pellicle within minutes on the tooth surface, followed by a biofilm after a longer period. Proteins including enzymes, lipids, and other components from saliva, form this acquired pellicle that modulates the attachment of bacteria to dental and epithelial surfaces [122]. Subsequently, the development of the pellicle mediates bacterial adherence to the non-shedding surfaces of sensors via various interactions, too [123]. Though, pellicle formation on sensor surfaces also influences measurements within the oral cavity. Biofouling processes can lower the performance of intraoral sensors by changing the permeability for the measured salivary substrates, which, combined with low metabolite concentrations, leads to poor sensitivity of the sensors [1, 28]. Besides, a reduced diffusion of metabolites might reduce the sensors’ response time [124]. The formation of a protein layer on the sensor surfaces may generate an insulating layer that potentially affects amperometric and potentiometric measurements. The pellicle contains several immobilized enzymes, e.g., amylase, peroxidase, and lysozyme [34], that potentially affect measurements performed with enzymatic biosensors. For example, peroxidase catalyzes the reduction of H_2_O_2,_ a common metabolite for biochemical sensors. Also, a detachment of enzymes from the sensor surface has to be avoided as well as leakage of potentially toxic sensor components [28]. Nanomaterials are often used to face this challenge and improve sensor sensitivity [1]. Oral sensors should be covered with protective permselective antimicrobial and anti-adherent coatings to circumvent the biofouling process caused by salivary proteins, e.g., mucins and proteolytic enzymes [1, 20, 28]. Appropriate coatings must be selected carefully to minimize or exclude electroactive interferences [28].

Amperometric or potentiometric biosensors are facile and promising approaches for validating salivary metabolites [25, 43, 75]. With different surface modifications incorporating specific ionophores or ion-dependent enzymes, kinetic measurements of calcium, phosphate, or fluoride can be carried out. However, for phosphate monitoring, the function of a sensor is highly depending on the kinetics between the immobilized enzyme and the electrode surface [125]. In detection setups based on an enzymatic reaction and H_2_O_2_ as a metabolite, a poor selectivity of the sensors is obtained when biological fluids are used as target sample. Likewise, the detection of O_2_ is limited by varying concentrations within the saliva sample, so challenges concerning the biosensor’s reproducibility arise [125].

However, unresolved challenges caused by interfering substances remain in monitoring lactate [43] or glucose [25] with amperometric enzymatic biosensors. For example, ascorbic or uric acid oxidize at the same specific voltage (in blood: + 600 mV) [103]. The effect has not been reported for substances other than blood yet. Hence, future research on in situ saliva sensor devices requires particular validation studies compared to blood [28]. Pre-treatments, e.g., tooth brushing, could be necessary to reduce interfering influences [103]. Still, by now, reports are lacking contributing on the relevance of adequate steps to reduce the effects of interfering substances in saliva. Here, an alternative approach could be a differential sensor with two identical cavities filled with enzyme/hydrogel and the other with hydrogel only. Individual signals could be subtracted easily, deriving the specific metabolite signal [103]. Furthermore, food or beverage intake could also cause interferences with the analyte observed [20].

**Fig. 3:**
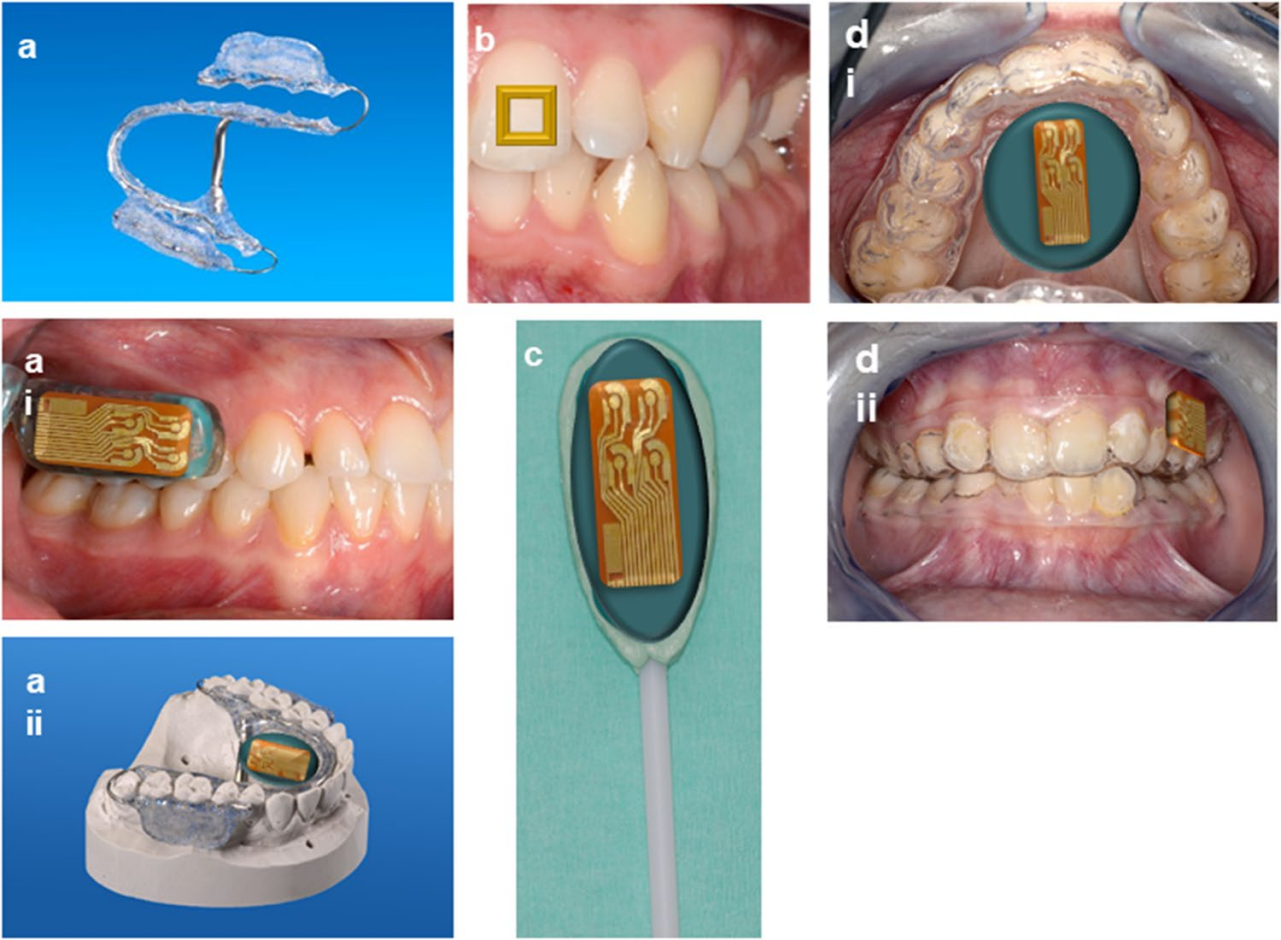
Schematic illustrations of devices for application of intraoral sensors. **a** Individualized orthodontic appliances. The embedding of the intraoral sensors is possible on the palate (i) or in the region of the upper molars (ii). **b** A dental tattoo fixed on an upper central incisor following Mannoor et al. [44] and Tseng et al. [45]. **c** Lollipop made of dental impression material for exposure in the cheek pocket with sensor attached. **d** Individualized vacuum-formed splints with sensor fixed on the palate (i) and the upper molars (ii) following Farella et al. [30] and Arakawa et al. [25]

Several possibilities exist for positioning the sensors in the easy accessible oral cavity. Ideally, suitable devices are as small as possible in order not to inhibit the salivary flow rate [30]. Oral devices, e.g., splints, pacifiers, dental tattoos, orthodontic appliances, or lollipops (Fig. 3a–d), are applicable forms with substantial comfort for the patient. Bulky mouthguard devices or broad electronic interfaces are not suited for regular use as glucose monitoring for diabetes patients [28, 43]. The oral instruments have to be anatomically correct to avoid gum bleeding by the sensor device due to contamination of the examined saliva [28]. Furthermore, saliva varies in its viscosity in different regions of the mouth, so the position of the sensing device has to be selected accordingly [24]. Research groups particularly focus on developing portable diagnostic tools [1]. A recent development toward miniaturization is a self-powered and minimally invasive glucose sensor using enzymatic fuel cells (EFCs), thereby eliminating an external power supply [126]. Wireless Bluetooth low energy (BLE) communication to transfer data from the sensor to a smartphone provides convenient handling by the user [25, 26, 41, 127].

Obeying the sensor materials’ safety and biocompatibility restrictions are critical tasks [28]. As the sensor stays in direct contact with the oral tissues, associating devices need adequate housings with preferably biocompatible materials [20]. The sensors can be covered effectively with materials commonly used and approved in dentistry, such as vinylpolysiloxane impression material [1]. Using potentially toxic or non-biocompatible materials, e.g., glutaraldehyde or nanomaterials, should be avoided or accordingly chosen with care [1, 67–70]. In addition, the stability of the sensors during intraoral operations and the possibility of proper cleaning and sterilization are essential facts to be considered [28]. Finally, the packaging of the device, especially of the electronic interface as well as the power supply, is of great importance to avoid shortcuts or excessively high currents [28].

New options for power supplies and data transfer, such as biofuel cells, solar cells, thermoelectric, piezoelectric, triboelectric, supercapacitor, or a combination thereof, have to be considered [28]. The best strategies regarding the challenge of wireless communication and long-distance data transmission between the sensor and the readout device in an energy-efficient modus include Bluetooth low energy (BLE) and near-field communication RFID (radio frequency identification) [20, 41]. Moreover, security issues must be addressed regarding patients’ health status when collecting and storing biomedical data in real time. Data must be protected from internal and external network attacks, so the risk of data misuse is minimized [28].

As saliva is a complex fluid, monitoring different metabolites simultaneously seems a promising approach. Most studies report on single analyte detection, which is insufficient when considering the overall health status [97]. An individual functionalization for each parameter follows the fabrication process of sensor foils. Likewise, multiplexed sensing platforms have been introduced to simultaneously measure saliva metabolites, such as glucose, lactate, cholesterol, and uric acid [128, 129]. Here, the precision of sensing devices, which simultaneously monitor the metabolites with spatially separated electrodes, can be improved by applying functionalized and non-functionalized electrodes to subtract potential interferences [97]. For certain metabolites, the introduction of new electrode materials that maximize the separation of their detection potentials can be a promising approach [130].

Until today, several research groups presented devices for real-time monitoring of different metabolites and ions in sweat and blood. However, intraoral real-time measurement of salivary metabolites with sensors still challenges researchers worldwide due to saliva’s complexity and dynamics [20].

## Conclusion

The oral cavity is the entrance gate to the digestive system and plays a key role in the diagnostics and treatment of different general diseases. From dental perspective, bacterial biofilm formation, taste and taste perception, and demineralization and remineralization occur in the wet oral environment. These processes are continuously part of ongoing research, but there is still limited knowledge on the kinetic and dynamic effects at the intraoral surfaces. The most relevant approach for shedding light on those mechanisms is monitoring the salivary biomarkers glucose, lactate, fluoride, calcium, phosphate, and pH with biosensors. These parameters have a high impact on the oral system and the dental surfaces. The concentration of these biomarkers can quickly change due to consumption of beverages, tooth cleaning, or sleeping. This literature review focusses on available sensor technology for monitoring the most relevant salivary biomarkers which have a high impact within the oral system and dental surfaces. The article highlights technological approaches as well as potential obstacles encountered within this very complex environment when monitoring these molecules, e.g., pellicle formation, interferences from salivary molecules or toxicity of materials.

By monitoring these dynamic biomarkers in situ and in real time with biosensors, new perspectives can be obtained about the complex and dynamic processes in the oral cavity, especially about the risk of caries development but also general diseases.

Promising technologies for electrochemical biosensing are still scarce, but the use of enzyme-based sensors seems an appropriate approach. No sensor that provides solid measurements beyond hours exists for any analyte than glucose*.* Saliva’s complex and dynamic structure is key challenge and should be addressed in the future. Therefore, studies that focus particularly on biofouling processes and interferential effects of the salivary matrix components on intraoral sensor and dental surfaces are required.
